# An Inveterate Head-Ach, Successfully Treated

**Published:** 1804-07-01

**Authors:** Wm. Lambe


### Medical and Physical Journal.

VOL. XII.] JULY 1, 1804. [NO. LXV.

Printed for R. PHILLIPS, by W. Thorne, Red Lion Court, Fleet Street, London.

### AN INVETERATE HEAD-ACH, SUCCESSFULLY TREATED. BY WM. LAMBE, M. D.

THE Rev. Mr. M—s, aged thirty-three, of melancholic temperament, had been afflicted with sick head-ach for sixteen years at the least. The paroxysms were of singular severity, lasting commonly for twenty-four, often for forty-eight hours, during which time he was incapable of any exertion, and generally, for a great part of it, was confined to his bed. These paroxysms were attended with great derangement of the stomach, eructation, frequently with sickness and vomiting. The attacks had recurred at irregular intervals: for the last year or two they had usually happened weekly; sometimes twice a week; for two months only, towards the end of last summer, he had intervals of two or three weeks; but in the autumn and beginning of winter they recurred as frequently and as severely as ever. At all times the bowels were much confined, the fæces hard and black, and the stomach perpetually oppressed with flatulence. From medicine, which had been applied under the best advice, he had received occasional benefit; but it never proved permanent, so that he was obliged to content himself with regulating the bowels, for which purpose the constant application of aperient medicines was indispensible. Except these distressing paroxysms, he could not be said to be out of health; the strength, sleep and appetite were natural; but the countenance was always of a dull and sickly paleness.

Having for some time been firmly of opinion, that constitutional diseases are produced by the *ingesta*, and among these, by contaminations of common water more than by all the others, I advised him to try the effect of a course of distilled water, confining himself to it entirely in every article, of which water is a principal ingredient. The proposed course could not be irksome to him, except as to to the trouble it would occasion, he never tasting malt liquor, and using wine very sparingly. The continuance of the wine I did not prohibit.

With this advice he has complied, and began the course at Christmas last. His diet, in other respects, has been exactly the same as usual; and the only medicine he has taken are some aperient pills, to which he has been accustomed for some years. In about three weeks, he found very sensible relief. The paroxysms of head-ach recurred as usual, but with much less violence; and ever since the disorder has continued to decline. For the last three months he has been attacked only three times, and then with so little severity, as not to interrupt his usual occupations. The general health has at the same time been materially improved. The flatulence is wholly removed, and the sickly paleness of the countenance has been succeeded by a general appearance of health, which he has not possessed for many years. I think it right to add, that this account has been in a great measure drawn up by himself.

It is impossible to account for so remarkable an effect, in consequence of a change apparently so simple, without suspecting common water to be impregnated, at least occasionally, with some matter of infinitely greater activity, than those which have hitherto been discovered in its composition. Such a matter I have in truth detected, and this detection occasioned me to recommend the course with so much confidence. I believe that the matter I allude to is a product of putrefaction, and that it is very generally diffused through the water applied to domestic uses. This matter I apprehend to be the principal source of the acrimonies, which have been so generally suspected to contaminate the animal fluids. I should have ventured to lay the grounds of these opinions before the public some time ago, had it not been for the extreme difficulties I have met with in the chemical investigations connected with this subject; difficulties, however, which I believe to be very nearly surmounted.

Though head-achs, such as that above described, are commonly free from danger, there are few complaints more unyielding to medicine. A regimen then, which will eradicate so rooted a malady, we may hope to find serviceable in other obstinate and intractable diseases. The instance I have adduced of essential benefit received by this treatment is by no means the only one which I possess. In due time the public shall be enabled to form their own opinion of its merit. In the mean time I am persuaded suaded that those, who choose to adopt it as an auxiliary to medicine, and in cases where medicine alone is inefficacious, will not be disappointed in any reasonable expectations they may form of its powers.

*King's Road, Bedford Row, May* 24, 1804.

